# Supplementary material for: Splice-Junction-Based Mapping of Alternative Isoforms in the Human Proteome
Source: Cell Rep. Author manuscript; Available in PMC 2020 Jan 15. (PMC6961840; doi:10.1016/j.celrep.2019.11.026)
Supplement: 2 [file NIHMS1546469-supplement-2.zip › DF1/PXD010154/Esophagus/Esophagus_19_SYNE3_SFTLMLRYNGPPPNTGGAGLGGAPGGPAASPAEGAHR.pdf]

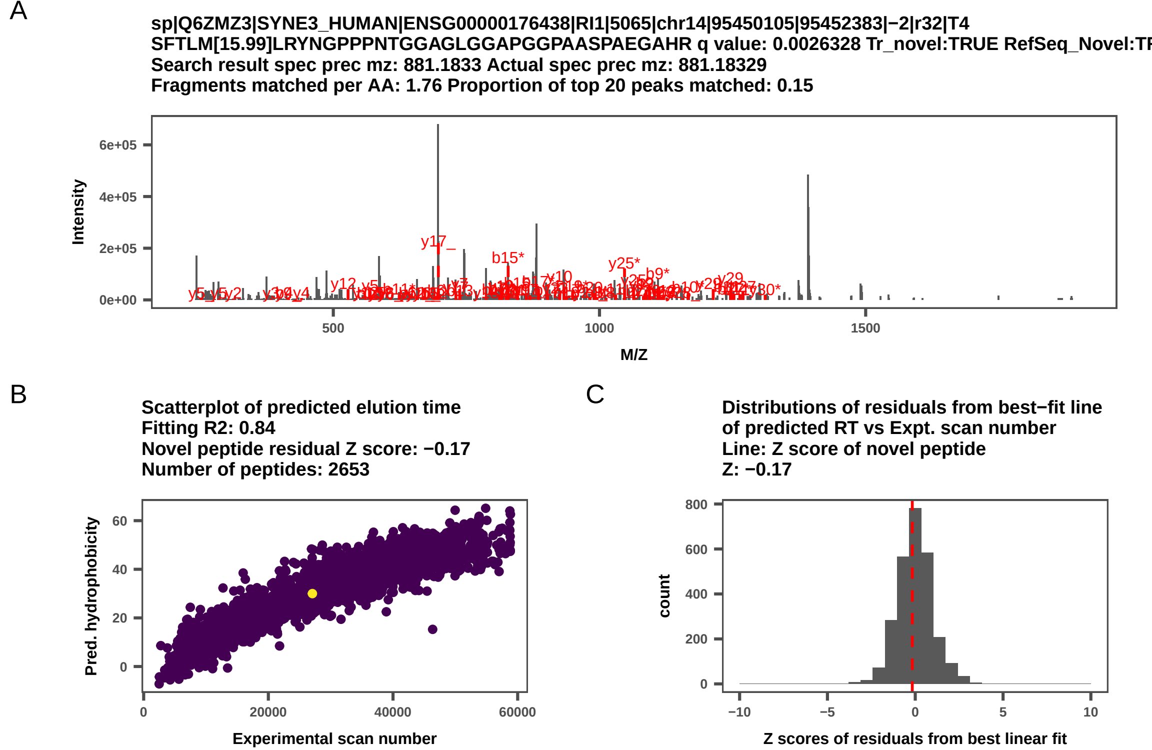
A
sp|Q6ZMZ3|SYNE3_HUMAN|ENSG00000176438|RI1|5065|chr14|95450105|95452383|−2|r32|T4
SFTLM[15.99]LRYNGPPPNTGGAGLGGAPGGPAASPAEGAHR q value: 0.0026328 Tr_novel:TRUE RefSeq_Novel:TF
Search result spec prec mz: 881.1833 Actual spec prec mz: 881.18329
Fragments matched per AA: 1.76 Proportion of top 20 peaks matched: 0.15
Intensity
6e+05
4e+05
2e+05
0e+00
y17_
b15*
y25*
b9*
500
1000
1500
M/Z
B
Scatterplot of predicted elution time
Fitting R2: 0.84
Novel peptide residual Z score: −0.17
Number of peptides: 2653
Pred. hydrophobicity
60
40
20
0
0
20000
40000
60000
Experimental scan number
C
Distributions of residuals from best−fit line
of predicted RT vs Expt. scan number
Line: Z score of novel peptide
Z: −0.17
count
800
600
400
200
0
−10
−5
0
5
10
Z scores of residuals from best linear fit
